# Impact of management on foliage-dwelling arthropods and dynamics within permanent pastures

**DOI:** 10.1038/s41598-019-46800-w

**Published:** 2019-07-31

**Authors:** Rocío Rosa García, Mariecia D. Fraser

**Affiliations:** 1 Servicio Regional de Investigación y Desarrollo Agroalimentario, Ctra. Oviedo s/n, 33300 Villaviciosa, Asturias Spain; 2 Pwllpeiran Upland Research Centre, Aberystwyth University, Cwmystwyth, Aberystwyth, Ceredigion SY23 4AB UK

**Keywords:** Agroecology, Grassland ecology

## Abstract

The restoration of biodiversity within previously improved grasslands is an important objective worldwide. In some areas farmers receive remuneration for using specific strategies but the environmental responses to them are still uncertain. This study explored the short and long-term impacts of sheep grazing and/or hay cutting on arthropod foliage communities and flora within Welsh upland permanent pastures (UK). We measured arthropod abundance and diversity plus sward surface height, flower numbers and percentage of forbs and grasses. Data were collected during summer; twice before hay cutting and once shortly after. Total arthropod abundance was higher in grazed plots (due to Symphypleona flourishing) and family richness in hay cut plots, but taxa-specific responses occurred. Short-term effects reflected phenological changes (e.g. in Symphypleona or Cantharidae) and arthropod reductions after hay cut, when mostly Diptera remained. Arthropod communities were more abundant and diverse in flower-rich and forb-dominated plots managed by hay cutting and by hay cutting with aftermath grazing, although certain groups flourished in grazed only grass-dominated plots. The two managements based on a hay cut provided more heterogeneous environmental conditions than other management treatments, and these supported more diverse arthropod communities. The results make a valuable addition to the evidence base on which to base future land use policy at a time when trade-offs between agricultural production and nature conservation are under scrutiny across Europe.

## Introduction

Intensification of agricultural practices across Europe has been linked to raised soil fertility, dominance of introduced grass species, and inappropriate management^1,2^; these are all factors that have contributed to the loss of biodiversity in grasslands^3,4^. The extensification of practices emerged as an alternative to restore or preserve grasslands in the late 1980s but acquired more prominence in the reform of the Common Agricultural Policy (CAP) in 2003, when the enrichment of biodiversity within previously improved grasslands became a goal for which farmers could receive remuneration. Recent communications from the EU Commission^5^ for the CAP beyond 2020 continue stressing the need to ensure that actively farmed areas are managed using practices that are beneficial to the environment. For permanent grasslands, both livestock grazing and hay cutting receive specific attention in the latest reforms. However, the effectiveness of the proposed measures is still debatable^6–8^, in part due to scarcity of long-term studies which have measured, under constant and controlled conditions, the effects of targeted practices on biodiversity^9^. Furthermore, in order to assess the adequacy of the different land management strategies for preserving habitats and or species, it is necessary to have a detailed understanding of how flora and fauna communities simultaneously evolve once management strategies change^10,11^.

Our knowledge regarding the suitability of different strategies to restore or preserve certain plant assemblages is greater than that for associated arthropod communities at the same scale and under the same management regimes^10,12^. Arthropods are a key component of grassland systems, making a significant contribution to biodiversity and ecosystem structure and function. They rely on the availability of resources (e.g. vegetation), which in turn varies greatly in response to management strategies^13^. Recent research effort in this area^9,14^ has greatly helped improve our understanding of related interactions^9^, but it has also emphasized the importance of site conditions and the specific objectives when determining a suitable strategy for each area^15^. The need for further research that examines short- and long-term responses to combined strategies simultaneously under real farmland conditions in order to evaluate the wider impact of management upon overall arthropod biodiversity has also been highlighted^16^.

This study explored the impacts of sheep grazing with and without hay cutting on flora and arthropod foliage communities within upland permanent pastures. Earlier work at the site had reported an increase in the number and diversity of forb species within plots managed for a hay cut compared to those that were grazed^17^. We hypothesized that such changes in floristic composition and structure would be reflected in greater arthropod abundance and diversity in plots with hay cut strategies. Long-term treatment responses of the fauna community and specific groups were hypothesized to differ with short-term within-season responses, particularly with regards to the substantial biomass loss associated with harvest which would concur with declines in arthropod abundance and diversity following hay cutting. Finally, we explored the differential contribution of specific sward characteristics in explaining the different arthropod communities.

## Results

A total of 62260 arthropods were collected and assigned to 13 orders and 44 families (Tables S1 and S2). The most abundant groups were Symphypleona, Diptera, Hemiptera and Hymenoptera (accounting for 33%, 23%, 21% and 14% of the catch counts respectively).

### Effect of management regime on sward characteristics

Sward height was similar between treatments during the first and second sampling periods (18th July (S1) and 16th August (S2), respectively), but drastically declined in the hay cut (H) and hay cut and aftermath grazing (HG) sites after the hay cut (28th August (S3)). The cut plots (H and HG) supported higher numbers of flowers (*P* < 0.05 and *P* < 0.001 respectively) and percentages of forbs (*P* < 0.001 both treatments) than grazed sites (sheep grazed (G) and control (CO)), but lower percentages of grasses (*P* < 0.001). The percentages of forbs and grasses had consistent inverse relationships (*P* < 0.001 and R^2^: 0.99 for S1–S2 and R^2^: 0.81 for S2–S3). During the first two periods, sward height correlated positively with the percentage of forbs (*P* < 0.001; R^2^: 0.50) and negatively with the percentage of grasses (*P* < 0.001; R^2^: 0.49). Later, in periods S2–S3, it only correlated (positively) with the percentage of grasses (*P* < 0.005 R^2^: 0.43). The number of flowers correlated positively with the forbs and negatively with the grasses during S1–S2 (*P* < 0.001; R^2^: 0.76 both) and also during S2–S3 (*P* < 0.001; R^2^: 0.68 for forbs, and *P* < 0.01; R^2^: 0.43 for grasses).

### Short-term (seasonal) and long-term (management regime) effects on arthropods

Total arthropod abundance changed over time (*P* < 0.001). The highest catches occurred in S2, whereas the lowest ones took place in S3 after the H and HG plots were cut (Table S1). Likewise, total abundance differed between treatments (*P* < 0.001). It was higher in CO than in the others and lower in G than in CO and H (*P* < 0.01). Lime addition had no effect on total abundance or diversity (number of families).

Comparing the first two periods (S1 and S2), the highest total abundance was recorded in CO due to the proliferation of Symphypleona in plant swards of this treatment. When this group was excluded from the analyses, the global abundance was higher in H and HG (*P* < 0.05) and G (*P* = 0.055) than in CO (Table S1). Fauna richness (number of families) differed between periods (higher in S2 than in S1) and treatments (*P* < 0.001), being higher in H than in CO (*P* < 0.05). The Shannon index was higher for G than CO (*P* < 0.05).

In addition to the global differences, the responses of the arthropods to the treatments were group-dependent: while Symphypleona (mostly Sminthuridae) abounded in grazed plots (especially in CO), other orders, such as Diptera and Coleoptera, flourished in hay plots. Coleoptera were more abundant in H plots than in the others (*P* < 0.001), largely due to the flower visitor family Cantharidae on these sites during S1 (Table 1). Diptera flourished in HG (*P* < 0.01) and H (*P* < 0.05) compared to G. Within this order, Syrphidae, was linked more closely with HG and H treatments than the other treatments (*P* < 0.001), whereas Tipulidae was not affected by sward management. Hemiptera globally proliferated more in H than in HG and G (*P* < 0.001) but family-dependent responses were observed. While Miridae and Aphididae flourished in H compared to G areas (*P* < 0.001), Cicadellidae peaked in G compared to H and HG (*P* < 0.05), and this last family was also favoured by lime addition (*P* < 0.001). In terms of spiders (Araneae) the response was mixed. Certain groups, such as Thomisidae, were more abundant in cut plots compared to grazed ones (*P* < 0.001 and *P* < 0.01 respectively), and also in sites with lime addition (*P* < 0.01). In contrast, the abundance of other families common to grasslands and agricultural land, such as Linyphiidae, did not differ between treatments.

**Table 1:** Results of RDA analyses for arthropod data collected on three occasions in 2013.

Env. var.	Cov.	Perm	%Axis	*F* fist/*F* all	*P* value
**Period 1–2**					
S*Treat	B	F,F	56.0	33.2/6.1	0.0020
Treat	S, B	F,N	14.5	5.4/2.2	0.0020
S	Treat, B	N,F	62.7	53.8	0.0020
S*Treat	S, PI, B	F,F	12.1	3.6/1.3	0.3820
Heig, flow, gras, forb	S, B,	F/F	12.8	4.9/3.2	0.0020
**Period 2–3**					
S*Treat	B	F,F	73.1	70.7/13.0	0.0020
Treat	S, B	F,N	33.3	16.0/3.3	0.0020
S	Treat, B	N,F	47.3	28.7	0.0020
S*Treat	S, PI, B	F,F	61.5	41.4/8.5	0.0040
Heig, flow, gras, forb	S, B,	F/F	59.5	49.9/16.9	0.0020

The environmental variables (Env. var.) used in the tests were: sampling period (S), Treatment (Treat) and the interaction of Time × Treatment (S × Treat), mean sward surface height (Heig), mean number of flowers (flow), percentage of grasses (gras) and percentage of forbs (forb). Covariables (Cov.): Block (B), sampling period (S), Treatment (Treat) and Plot Identifier (PI). The permutation scheme (Perm) on the whole plot level/ split plot level was: freely (F) or no permutation (N). %Axis: Cumulative percentage of variance of species data explained by Axis 1. F first, F all: values of F on the first ordination axis and all constrained axes respectively. P value: P values on all the ordination axes. Results of non-standardized tests are provided.

### Responses to hay cutting

Total arthropod abundance changed between periods S2 and S3 (*P* < 0.001). Abundance in G sites was steady whereas in CO it increased due to Sminthuridae proliferation. In contrast, abundance dropped sharply in H and HG in S3 (with no differences between these treatments) compared to the grazed sites (*P* < 0.001). Fauna richness and Shannon index values also fell for H and HG plots (where mostly Diptera remained after the cut) compared to grazed ones (*P* < 0.001). Within the grazed plots no differences in diversity indices were observed over these time periods.

### Responses of arthropod foliage assemblages to sward management and related plant variables

Multivariate analyses were used to test the effects of treatment on arthropod community composition. These differed between periods (comparing S1 and S2) and treatments (*P* < 0.01, for all axes; Table 1).

**Figure 1:**
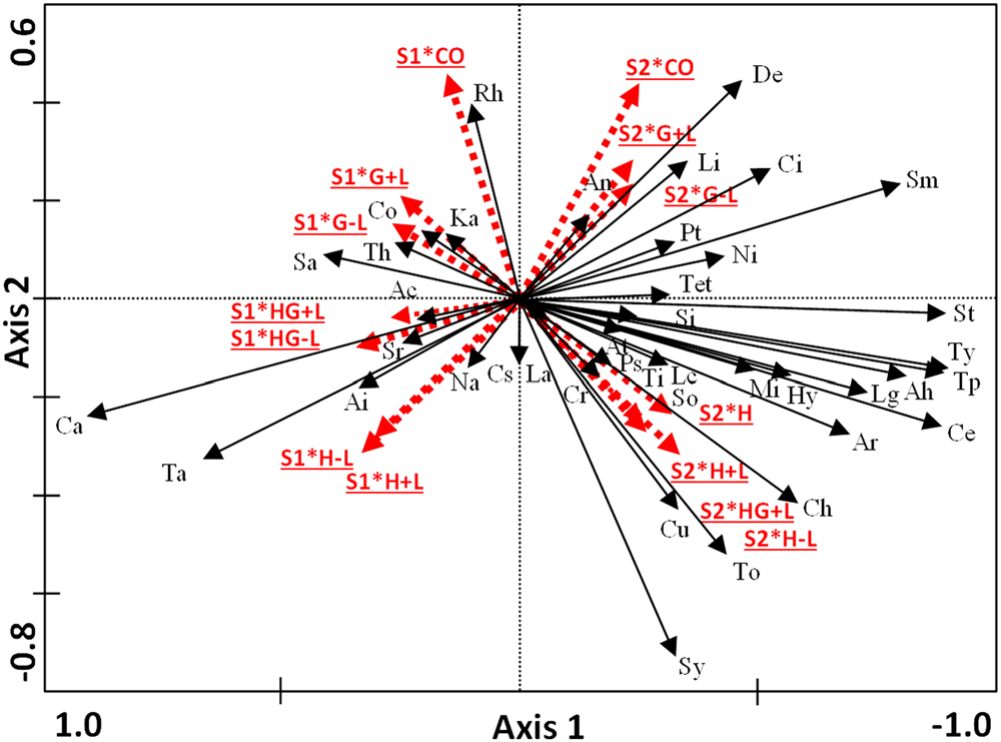
Biplot of Season × Treat interaction for a RDA analysis which explored the responses of arthropod community during the first two periods. Dotted red arrows: treatments (CO, G, HG, H) in a period (S1, S2, S3). Black arrows: arthropod families (see Table S2 for abbreviations). Analyses performed for a repeated measures design. Blocks included as covariables. Non-standardization applied.

The gradient along axis 1 of Fig. 1 reflects differences in community composition between the first period (right side) and the second (left side). It shows the seasonal fluctuations of certain species (i.e. Cantharidae peaked in July versus Tipulidae in August). Axis 2 defines the preferences of the taxa for different grazing treatments. Thus, while families such as Delphacidae, Sminthuridae and Cicadellidae flourished in the grazing treatments, a greater number of arthropods were more abundant where a hay cutting treatment was applied (bottom), especially during S2. Since the period x treatment interaction was not significant, the effect of period and treatment were additive.

**Figure 2:**
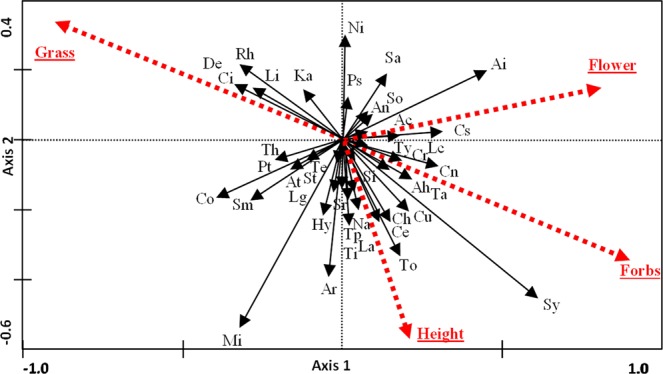
Biplot of RDA analysis which explored the relationships between arthropod community and the environmental variables during the first and second periods. Dotted red arrows are environmental variables: mean sward height (Height), mean number of flowers (Flowers), percentage of forbs (Forbs) and percentage of grasses (Grass). Black lines: arthropod families (see Table S2 for abbreviations). Repeated measures design with blocks and periods as covariables. Non-standardization applied.

Vegetation parameters explained a significant percentage of the variance for the arthropod assemblages (*P* < 0.01 for all axes; Table 1) during the periods S1 and S2. The automatic forward selection of the four parameters included as environmental variables was significant for sward height (*P*: 0.002; F: 5.14) and the number of flowers (*P*: 0.002; F: 4.25). Figure 2 shows the response of particular arthropod groups to the different environmental variables. Many groups preferred swards with a higher percentage of forbs and number of flowers (the right side). A smaller group was collected in higher densities in the areas dominated by grasses (towards the left).

**Figure 3:**
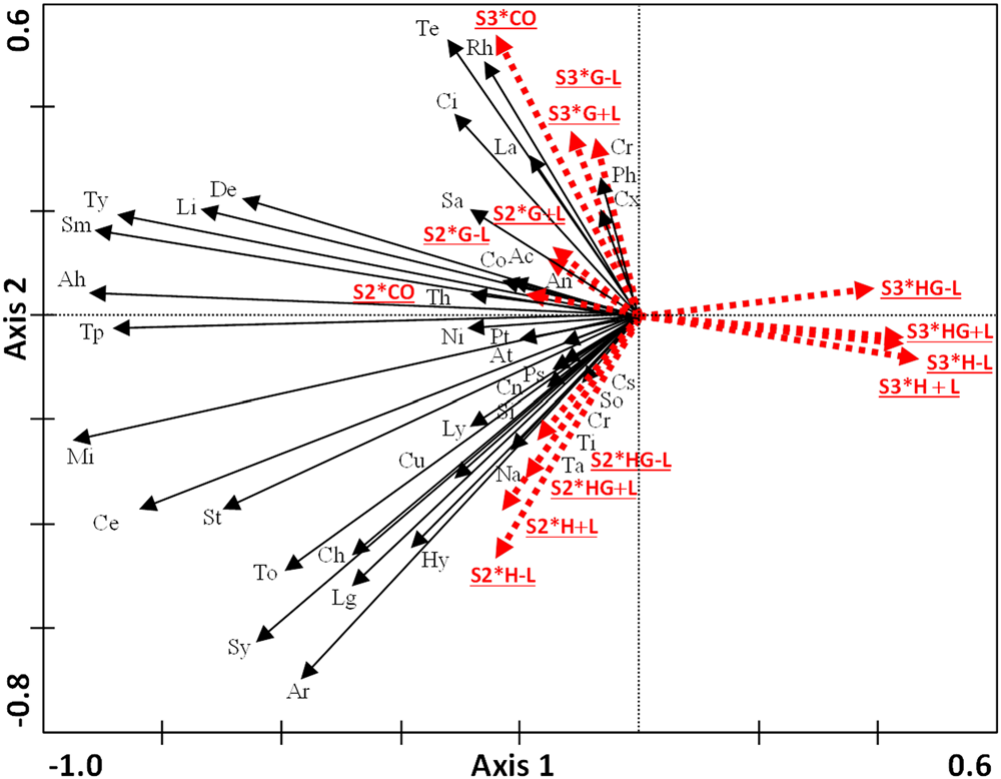
Biplot of Season*Treatment interaction (Season*Treat) for a RDA analysis which explored the responses of arthropod foliage community during the second and third periods. The dotted red arrows represent the treatments (CO, G, HG, H) in a period (S2, S3). Black lines: arthropod families (see Table S2 for abbreviations). Analyses were performed for a repeated measures design. Blocks were included as covariables. Non-standardization applied.

The analysis of the changes in community composition between S2 and S3 revealed significant differences between periods and among treatments, as well as significant interactions between these (Table 1). The most drastic changes in community composition occurred after hay cut (H and HG, Fig. 3). While the abundance of a wide variety of taxa was positively associated with these treatments during S2, the situation changed in the short-term and these treatments appear clearly separated from the rest and with less diverse communities associated with them. Axis 2 defines specific responses of different taxa to the grazing only treatments (CO and G) compared to the rest (H and HG).

**Figure 4:**
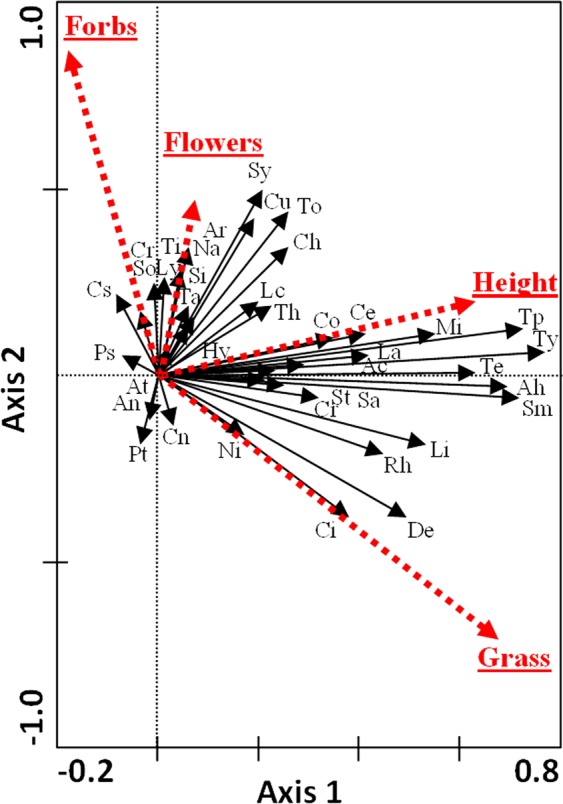
Biplot of RDA analysis which explored the relationships between arthropod foliage community and the environmental variables during the second and third periods. The dotted red arrows represent the environmental variables: mean sward height (Height), mean number of flowers (Flowers), percentage of forbs (Forbs) and percentage of grasses (Grass). Black lines: arthropod families (see Table S2 for abbreviations). Analyses were performed for a repeated measures design. Blocks and periods included as covariables. Non-standardization applied.

Vegetation parameters also explained a significant percentage of the variance in arthropod assemblages for periods S2 and S3 (*P* < 0.01 for all axes; Table 1). These parameters included sward height (*P*: 0.002; F: 42.31), the percentage of grasses (*P*: 0.002; F: 8.74), the number of flowers (*P*: 0.018; F: 2.24) and the percentage of forbs (*P*: 0.054; F: 1.85). The number of arthropod taxa whose abundance was positively correlated with increases in the percentage of grasses was low whereas more taxa abounded in plots with higher sward heights (right side of Axis 1, Fig. 4) and higher percentage of forbs (top part of Axis 2). The preference of certain groups for certain conditions persisted, e.g. Syrphidae and Thomisidae for areas with greater number of flowers and various sap sucking insects for areas with higher percentages of grasses.

## Discussion

This study confirmed that specific groups of arthropods associated with sward different management strategies, and their responses, varied at different temporal scales. The differential responses may be explained by their different life strategies, ecological needs and sensitivity to the treatments^14–16,18^. Overall, the strategies which included hay cutting were associated with a higher diversity of arthropod groups than those treatments in which swards were only grazed, but they suffered drastic losses in the short term (just after cutting). A meta-analysis based on 35 relevant studies on grazing and annual mowing, including short- and long-term experiments, detected positive short-term effects of grazing and long-term effects of mowing^14^. This last strategy favours greater diversity of beetles and butterflies both alone or with cattle grazing^16^.

The short-term responses of arthropod groups depend on multiple factors including their phenology, mobility, and sensitivity to the alteration of habitat characteristics such as changes in abundance of prey/food plants, foliage habitat structure and shifts in microclimate conditions. In this study the seasonal fluctuations in abundances were taxa-dependent; e.g. Cantharidae peaked in S1 to decline later on, whereas Sminthuridae increased after S2. At the same time both taxa responded consistently to the treatments: Cantharidae flourished in cut areas (H and HG) and Sminthuridae in the control (CO). Cantharidae eat pollen and nectar on Apiaceae or Asteraceae^19^ whereas Sminthuridae, which feed on living plant tissue such as young grass, prefer greater soil moisture and peak in numbers in summer^20^. Mobile groups such as Diptera dominated after a hay cut, once the availability of resources drastically changed, whereas flightless and less mobile groups, such as spiders, mostly disappeared. Vegetation provides food, shelter and/or structure for spiders and their herbivorous preys, and both groups responded negatively to vegetation removal. Negative responses by less mobile, flightless taxa after mowing have also been reported by Mazalová *et al*.^16^.

Plant variables were greatly influenced by the management strategies and different plant variables associated with different fauna communities. Previous studies also detected links between specific plants and arthropod communities in crops^21^, and a positive relationship between pollinators and plant species richness in cut grasslands^22^. Global responses of the wider community of arthropods to changes in plant community composition under different land management strategies (including mowing/fertilization regimes) have been detected^21^ and linked to changes in the availability of key plant resources^23–25^, including the abundance and richness of grasses or forbs^22^. This supports the conclusion that small-scale plant community characteristics must be taken into account for a better understanding of arthropods’ response to the farming system^21^.

In our study, the abundance of pollinators, such as hoverflies or bees, was higher in the areas with higher percentages of forbs and higher number of flowers, in agreement with previous studies which concluded that high numbers of flowers and percentages of forbs are potentially favorable to pollinators^26,27^. A combination of management strategies which contribute to the conservation of both fauna and flora communities is essential where flower-visiting insects are crucial for the pollination of plant species such as legumes^28^.

Diet selection by grazing livestock results in more selective defoliation compared to mechanical cutting and, over time, this can lead to a change in sward botanical composition. In our study, sheep grazing was associated with fewer short-term effects on arthropod taxa (except for Sminthuridae), likely because neither plant composition nor resource availability changed notably between S2 and S3 in the grazed sites. The long-term impact differed depending on whether grazing was combined with hay cutting or not. Thus, hay cut and aftermath grazing favoured more diverse plant and arthropod communities compared to grazing only. The dominance of a reduced group of arthropods, such as Cicadellidae or Collembola, in grazed than in ungrazed plots could be related to the greater homogeneity of the canopy displaying a lower variety of environmental conditions. Additionally, the dominance of mostly anemophilous plant species may explain the reduced pollinator community diversity in the grazed sites. The findings concur with those from a previous study, which found that after six years of sheep grazing in an abandoned meadow the proportion of dominant herbs decreased as grasses increased in the grazed plots^29^. However, further research is required to explore the comparative impact of using less selective grazing animals, such as cattle^30^, to graze permanent pastures.

## Implications

Habitat and species loss in EU upland permanent pastures is on-going. The relative effects of different strategies are highly variable and still poorly quantified. The CAP is under constant review to search for sustainable prescriptions and adjust the associated policies. The current findings relate to a grassland type which would be considered ecologically poor; long-term reseeded improved pasture that had historically received regular applications of inorganic fertilizer to maintain *Lolium perenne* dominance. As such, this study provides evidence that management practices that are now in decline in the EU Atlantic region, such as hay-making, have potential to deliver multiple ecosystem services from floristically challenged swards, and should be maintained. A number of those services depend on the conservation of plant and insect richness (including pollinators) which contribute to increasing productivity in the absence of fertilizers, as well as the storage of greater densities of carbon while producing less nitrous oxide^31^. This study provided detailed information on the extent to which different arthropod groups respond to alternative management options. In practice, a combination of strategies (including sequential mowing of land units and appropriate grazing regimes) within a mosaic at landscape scale could maximize the delivery of biodiversity and associated services.

## Conclusions

This study, based on an improved upland permanent pastures, revealed that hay cutting alone or combined with aftermath grazing are not only good strategies to maintain more diverse plants swards with greater numbers of flowers than grazing alone, but they also promoted local arthropod biodiversity. Whilst mowing led to significant reductions in arthropod abundance and diversity in the short term, this management option provided a wider variety of resources in the long term and thus supported more diverse arthropod communities, including a higher abundance and diversity of pollinators. In contrast, sheep grazing generated a less diverse, grass-dominated sward suitable for a narrower range of arthropod taxa. Therefore, mowing has strong potential for the conservation of biodiversity and other ecosystem services and the conservation of such traditional practices deserves further attention.

## Methods

### Study site

The experiment was conducted using the Brignant long-term plots established in 1995 at the Pwllpeiran Upland Research Centre, Ceredigion, Wales, UK (52°21′55″N, 3°49′49″W). These are located at an altitude of 310 m a.s.l., on free-draining typical brown podzolic soils and receive a mean annual rainfall of approximately 1850 mm, with minimum and maximum air temperatures of 5.2 °C and 11.9 °C respectively. The permanent pasture was last ploughed and reseeded in 1973, and until the experimental regimes were imposed, had received regular inputs of fertilizer and lime. At the time of establishment, the site was dominated by grass species (particularly the sown species *Lolium perenne*, at 58% cover) and low frequency of desirable forb species.

### Experimental design

The Brignant long-term plots are arranged in a randomized block design with three blocks and seven grassland management regimes: sheep grazing, with (GL+) and without (GL−) lime application; hay cutting only, with (HL+) and without (HL−) lime application; and hay cutting followed by aftermath sheep grazing, with (HGL+) and without (HGL−) lime application. Control (CO) plots continuing the previous management (i.e. limed, fertilised and continually grazed by sheep) were also included in each block. They receive an annual application of 60 kg N fertilizer and 30 kg P fertilizer ha^−1^. All the lime treatments received a single application in 1998 to maintain a soil pH of 6.0. Treatments were imposed on plots of 0.08 ha (hay cut only) or 0.15 ha (cut and grazed) in size. The plots are stocked with sheep (usually Welsh Hill Speckled Faced yearlings) with numbers adjusted to maintain a sward surface height of 4–6 cm. There is no spring grazing of the HGL+ and HGL− treatments to allow the colonizing forb species an opportunity to establish. The HL and HGL plots have a single annual hay harvest in July, mechanically cut and baled during appropriate weather. The HGL+ and HGL− plots are subsequently restocked until September.

### Arthropod assessments

Arthropods were sampled by timed sweep netting on three occasions in 2013, two before and the last one after hay cut: 18th July (S1), 16th August (S2) and 28th August (S3). Within each plot, one sweep was taken after each of 50 consecutive strides along a linear random transect using a 40 cm diameter sweep net. Three or two transects were established in each plot (to adjust sampling effort to plot size), at a minimum of 3 m away from the plot fences to prevent border effects. Sweep net collections provide a rapid and easily standardized protocol and the sampling effort in this experiment was considered adequate to measure the local arthropod communities properly and to be able to compare them between treatments. The specimens from each transect were preserved in vials containing 70% ethanol. The adult arthropods were identified to order and family level (for the orders Coleoptera, Hemiptera and Araneae). The statistical analyses were performed on the average number of individuals/taxa per transect, with three replicates (plots) per treatment and three periods of data.

### Botanical assessments

Sward surface height, the number of flowers, and the percentage of forbs and grasses were recorded at each arthropod sampling event. These parameters are considered good indicators to address the differences between pastures and they have also been recognized as important ones for describing the habitat characteristics for the fauna. A total of 40 random measurements of the sward height were performed with a sward stick in each plot. Percentage cover of the main vegetation components (forbs, grasses, moss and bare ground) was estimated and the number of flowers quadrat was recorded within a 0.5 m × 0.5 m at 10 random locations across each plot.

### Statistical analyses

A mixed model procedure for repeated measures was used to examine the differences in plant parameters, as well as in the abundance and diversity of arthropods, between management regimes, and over time, for a complete block design with 21 plots. Block, sward treatment (four cutting/grazing treatments), liming treatment (with or without the application of lime) and the interaction of sward and liming treatments were included in the model as fixed factors. The meaned arthropod numbers for each plot were log transformed and plot vegetation cover data was arcsine transformed when necessary to meet assumptions of normality and homocedasticity. The finite-sample corrected Akaike information criterion (AIC)^32^ was used to select the models which best fitted the covariance structure for each dependent variable. Bonferroni multiple-comparison tests were used for post-hoc comparisons where significant treatment effects were identified. Partial correlations (controlling for block and period) were conducted to assess the relationships between flora variables. All the analyses were performed using SAS System software^33^.

Redundancy analysis (RDA) in the CANOCO program^33^ (version 4.5) was used to evaluate the differences in arthropod community composition between treatments and sampling periods. The adequacy of this method was confirmed by a preliminary detrended correspondence analysis (DCA) which yielded short axes lengths of < 3 SD^35^. Various combinations of environmental (explanatory) variables and covariables were used, together with specific split-plot permutation schemes, to test the particular effects on arthropod community composition in a comparable way to repeated measures ANOVA^34^. Monte Carlo permutation tests with 499 permutations were then used to determine significant effects of those explanatory variables^34^. The split plots in the permutation schemes were the pooled observations within each whole plot. Plot identifiers (coded as dummy variables) were used as covariables when the influence of the three treatments over time in arthropod fauna assemblages was tested. All analyses were performed on log transformed arthropod data and centering by species. A figure was included to explain the effects of period × treatment interactions as explanatory variables. This corresponds to pooled main and interaction effects in ANOVA and provides an indication of similarity of individual treatments in individual periods.

RDA was also used to calculate the variability in arthropod species abundance accounted for by selected explanatory variables (plant height, the number of flowers and percentage cover of grasses and forbs) coded as quantitative variables. A reduced model was generated including each environmental variable during the forward selection and evaluating its statistical significance by the F-ratio based on the trace and 499 Monte Carlo permutations^35^. The plot was the statistical unit in all analyses.

## Supplementary information

Tables 1 and 2 Supplementary information

## Data Availability

The datasets generated during and/or analyzed during the current study are available from the corresponding author on reasonable request.
