# Supplementary material for: Impact of management on foliage-dwelling arthropods and dynamics within permanent pastures
Source: Sci Rep. 2019 Jul 31;9:11090. doi: 10.1038/s41598-019-46800-w (PMC6668424; doi:10.1038/s41598-019-46800-w)
Supplement: Supplementary file 1 — Tables 1 and 2 Supplementary information [file 41598_2019_46800_MOESM1_ESM.docx]

**TITLE: Impact of management on foliage-dwelling arthropods and dynamics within permanent pastures**

Authors: Rocío Rosa García^a,*^, Mariecia D. Fraser^b^

^a^ Servicio Regional de Investigación y Desarrollo Agroalimentario, Ctra. Oviedo s/n, 33300, Villaviciosa, Asturias, Spain. E-mail: rocior@serida.org

^b^ Pwllpeiran Upland Research Centre, Aberystwyth University, Cwmystwyth, Aberystwyth, Ceredigion SY23 4AB, UK. E-mail: mdf@aber.ac.uk

Corresponding author: Rocío Rosa García

**Table S1** Mean number of foliage arthropods per transect and period for each grazing/cutting period

|  | **PERIOD 1** | | | | | | | | | | | | | |
| --- | --- | --- | --- | --- | --- | --- | --- | --- | --- | --- | --- | --- | --- | --- |
|  | CO | | HG+L | | HG-L | | G+L | | G-L | | H+L | | H-L | |
|  | Mean | SEM | Mean | SEM | Mean | SEM | Mean | SEM | Mean | SEM | Mean | SEM | Mean | SEM |
| **FLORA** |  |  |  |  |  |  |  |  |  |  |  |  |  |  |
| Sward height | 38.58 | 6.68 | 40.50 | 9.01 | 35.82 | 5.26 | 36.12 | 6.34 | 35.87 | 9.42 | 43.74 | 5.12 | 43.87 | 5.76 |
| Nºflowers | 0.00 | 0.00 | 7.60 | 0.99 | 7.07 | 2.76 | 0.87 | 0.67 | 0.70 | 0.35 | 5.53 | 1.85 | 7.03 | 2.00 |
| %Grass | 98.37 | 1.28 | 58.63 | 2.42 | 39.10 | 7.40 | 93.60 | 2.39 | 95.63 | 1.18 | 41.30 | 3.70 | 47.93 | 4.31 |
| %Forbs | 1.33 | 1.03 | 40.97 | 2.32 | 59.77 | 6.82 | 4.77 | 1.48 | 1.33 | 0.28 | 58.17 | 3.84 | 51.77 | 4.27 |
| **FAUNA** |  |  |  |  |  |  |  |  |  |  |  |  |  |  |
| Total abundance | 225.67 | 28.53 | 228.33 | 35.50 | 254.11 | 14.69 | 160.56 | 7.39 | 222.00 | 51.68 | 247.17 | 5.42 | 237.33 | 36.13 |
| Abundance excluding Symphypleona | 157.00 | 8.41 | 204.89 | 47.73 | 250.00 | 16.84 | 141.11 | 8.19 | 208.11 | 51.32 | 244.67 | 4.78 | 232.17 | 35.28 |
| Family richness | 14.00 | 2.08 | 13.67 | 1.86 | 14.67 | 0.88 | 14.33 | 0.88 | 17.00 | 0.58 | 14.00 | 1.73 | 15.00 | 1.15 |
| Shannon index | 1.31 | 0.10 | 1.70 | 0.16 | 1.97 | 0.05 | 1.36 | 0.15 | 1.85 | 0.10 | 1.57 | 0.10 | 1.66 | 0.17 |
| O. Araneae | 9.56 | 7.59 | 3.67 | 2.52 | 3.56 | 0.97 | 6.89 | 3.09 | 5.67 | 0.96 | 1.50 | 0.76 | 2.67 | 0.44 |
| Fam. Linyphiidae | 9.00 | 7.53 | 3.33 | 2.67 | 2.67 | 1.00 | 5.22 | 3.42 | 2.67 | 1.39 | 0.50 | 0.29 | 1.17 | 0.93 |
| O. Coleoptera | 2.33 | 1.39 | 5.22 | 1.28 | 5.22 | 1.18 | 2.67 | 0.84 | 5.33 | 2.33 | 17.33 | 6.29 | 13.00 | 1.15 |
| Fam. Cantharidae | 2.11 | 1.31 | 5.00 | 1.17 | 5.22 | 1.18 | 1.89 | 0.56 | 4.56 | 2.30 | 16.67 | 6.41 | 12.50 | 1.04 |
| O. Diptera | 39.11 | 6.84 | 102.78 | 34.44 | 178.78 | 37.24 | 43.56 | 6.14 | 33.00 | 9.96 | 89.83 | 24.57 | 70.17 | 13.41 |
| Fam. Syrphidae | 1.00 | 0.38 | 2.89 | 0.59 | 2.56 | 0.73 | 0.44 | 0.44 | 0.78 | 0.22 | 2.17 | 0.60 | 2.33 | 0.44 |
| Fam. Tipulidae | 1.44 | 0.78 | 0.44 | 0.22 | 0.56 | 0.11 | 0.44 | 0.22 | 0.33 | 0.19 | 1.17 | 0.93 | 0.50 | 0.29 |
| O. Hemiptera | 76.89 | 11.53 | 37.78 | 9.46 | 24.44 | 7.82 | 55.78 | 6.34 | 40.78 | 8.46 | 63.00 | 23.46 | 60.00 | 7.97 |
| Fam. Cicadellidae | 0.22 | 0.11 | 0.56 | 0.22 | 0.44 | 0.29 | 0.44 | 0.22 | 2.44 | 0.40 | 0.33 | 0.17 | 0.50 | 0.29 |
| Fam. Miridae | 66.78 | 13.93 | 22.67 | 9.08 | 13.78 | 5.90 | 47.11 | 8.78 | 31.56 | 8.42 | 47.83 | 20.64 | 39.00 | 14.37 |
| Fam. Delphacidae | 1.11 | 0.29 | 0.56 | 0.40 | 0.33 | 0.00 | 2.00 | 0.77 | 2.11 | 0.97 | 1.17 | 0.67 | 1.00 | 0.50 |
| Fam. Aphididae | 8.44 | 2.70 | 13.00 | 1.76 | 9.67 | 2.33 | 6.00 | 3.02 | 4.11 | 1.82 | 12.83 | 3.71 | 18.17 | 6.84 |
| O. Hymenoptera | 28.11 | 4.56 | 53.11 | 15.30 | 34.78 | 17.35 | 29.89 | 9.10 | 118.67 | 53.85 | 71.33 | 17.31 | 84.17 | 32.36 |
| O. Symphypleona | 68.67 | 22.52 | 23.44 | 16.39 | 4.11 | 2.16 | 19.44 | 11.46 | 13.89 | 10.13 | 2.50 | 0.76 | 5.17 | 1.86 |

**Table S1** Mean number of foliage arthropods per transect and period for each grazing/cutting period. Continued

|  | **PERIOD 2** | | | | | | | | | | | | | |
| --- | --- | --- | --- | --- | --- | --- | --- | --- | --- | --- | --- | --- | --- | --- |
|  | CO | | HG+L | | G-L | | G+L | | H+L | | HG-L | | H-L | |
|  | Mean | SEM | Mean | SEM | Mean | SEM | Mean | SEM | Mean | SEM | Mean | SEM | Mean | SEM |
| **FLORA** |  |  |  |  |  |  |  |  |  |  |  |  |  |  |
| Sward height | 36.43 | 2.03 | 49.11 | 3.53 | 37.66 | 5.54 | 37.19 | 3.92 | 51.28 | 3.72 | 41.90 | 4.13 | 48.89 | 4.11 |
| Nºflowers | 0.00 | 0.00 | 1.97 | 0.81 | 0.07 | 0.07 | 0.07 | 0.07 | 0.53 | 0.34 | 3.33 | 1.45 | 0.67 | 0.17 |
| %Grass | 98.70 | 0.25 | 59.30 | 2.93 | 94.77 | 0.84 | 93.73 | 3.62 | 55.50 | 3.25 | 40.67 | 8.48 | 65.00 | 1.15 |
| %Forbs | 1.30 | 0.25 | 40.57 | 3.05 | 4.68 | 0.82 | 5.86 | 3.75 | 44.50 | 3.25 | 57.97 | 8.02 | 34.50 | 0.76 |
| **FAUNA** |  |  |  |  |  |  |  |  |  |  |  |  |  |  |
| Total abundance | 674.56 | 104.49 | 462.56 | 82.59 | 373.11 | 66.27 | 428.44 | 195.93 | 541.17 | 113.75 | 512.78 | 67.06 | 515.50 | 44.93 |
| Abundance without Symphypleona | 235.00 | 29.26 | 356.33 | 34.35 | 275.22 | 51.80 | 310.78 | 118.07 | 397.33 | 106.66 | 388.89 | 38.06 | 366.50 | 15.95 |
| Family richness | 17.00 | 1.00 | 21.00 | 0.58 | 20.67 | 2.73 | 18.67 | 0.88 | 19.33 | 0.88 | 21.00 | 1.53 | 18.33 | 0.88 |
| Shannon index | 1.01 | 0.27 | 1.83 | 0.18 | 1.73 | 0.16 | 1.80 | 0.08 | 1.60 | 0.07 | 1.71 | 0.12 | 1.67 | 0.04 |
| O. Araneae | 5.00 | 1.02 | 7.78 | 1.54 | 9.67 | 2.52 | 19.22 | 14.60 | 8.50 | 2.78 | 8.44 | 1.54 | 14.50 | 3.46 |
| Fam. Linyphiidae | 3.33 | 1.07 | 3.67 | 2.36 | 7.89 | 3.35 | 18.11 | 14.18 | 3.50 | 1.26 | 2.22 | 0.48 | 7.67 | 3.81 |
| O. Coleoptera | 5.33 | 1.50 | 6.56 | 1.16 | 13.00 | 10.67 | 4.89 | 1.57 | 5.33 | 0.83 | 5.56 | 1.95 | 9.67 | 1.59 |
| Fam. Cantharidae | 0.11 | 0.11 | 0.11 | 0.11 | 0.00 | 0.00 | 0.00 | 0.00 | 0.00 | 0.00 | 0.00 | 0.00 | 0.00 | 0.00 |
| O. Diptera | 47.44 | 7.55 | 86.78 | 14.90 | 37.67 | 8.02 | 46.78 | 15.83 | 82.17 | 13.58 | 164.56 | 48.59 | 70.67 | 13.97 |
| Fam. Syrphidae | 0.89 | 0.29 | 6.11 | 0.11 | 0.78 | 0.11 | 1.00 | 0.38 | 8.67 | 3.35 | 4.33 | 1.07 | 4.33 | 0.88 |
| Fam. Tipulidae | 6.22 | 1.56 | 10.67 | 3.48 | 8.00 | 3.21 | 5.67 | 0.51 | 20.00 | 7.82 | 7.89 | 3.66 | 16.00 | 7.21 |
| O. Hemiptera | 105.67 | 13.64 | 149.11 | 43.43 | 126.44 | 22.16 | 127.22 | 56.95 | 225.50 | 98.93 | 106.22 | 10.50 | 194.83 | 29.48 |
| Fam. Cicadellidae | 2.11 | 0.22 | 1.78 | 0.68 | 4.11 | 1.87 | 5.00 | 2.27 | 3.00 | 1.32 | 0.67 | 0.19 | 0.83 | 0.33 |
| Fam. Miridae | 47.89 | 12.37 | 99.78 | 33.90 | 33.78 | 4.36 | 39.44 | 14.51 | 123.50 | 71.78 | 59.22 | 14.51 | 114.33 | 16.31 |
| Fam. Delphacidae | 5.44 | 1.35 | 1.89 | 0.11 | 6.78 | 4.78 | 9.22 | 4.33 | 2.17 | 0.60 | 1.22 | 0.40 | 1.00 | 0.29 |
| Fam. Aphididae | 44.78 | 2.80 | 40.11 | 10.34 | 78.44 | 15.14 | 69.56 | 37.13 | 86.67 | 31.35 | 39.44 | 5.17 | 69.17 | 20.64 |
| O. Hymenoptera | 58.67 | 5.74 | 72.78 | 9.03 | 59.00 | 10.84 | 67.67 | 23.31 | 57.00 | 10.25 | 59.11 | 3.58 | 54.00 | 5.75 |
| O. Symphypleona | 439.56 | 132.47 | 106.22 | 49.10 | 97.89 | 56.05 | 117.67 | 83.89 | 143.83 | 42.77 | 123.89 | 52.99 | 149.00 | 45.24 |

**Table S1** Mean number of foliage arthropods per transect and period for each grazing/cutting period. Continued.

|  | **PERIOD 3** | | | | | | | | | | | | | |
| --- | --- | --- | --- | --- | --- | --- | --- | --- | --- | --- | --- | --- | --- | --- |
|  | CO | | HG+L | | G-L | | G+L | | H+L | | HG-L | | H-L | |
|  | Mean | SEM | Mean | SEM | Mean | SEM | Mean | SEM | Mean | SEM | Mean | SEM | Mean | SEM |
| **FLORA** |  |  |  |  |  |  |  |  |  |  |  |  |  |  |
| Sward height | 32.46 | 2.17 | 6.43 | 0.22 | 35.17 | 3.00 | 32.88 | 1.79 | 5.53 | 0.36 | 5.34 | 0.68 | 5.76 | 0.09 |
| Nºflowers | 0.17 | 0.12 | 0.00 | 0.00 | 0.23 | 0.13 | 0.50 | 0.23 | 0.00 | 0.00 | 0.00 | 0.00 | 0.00 | 0.00 |
| %Grass | 94.40 | 2.74 | 61.50 | 10.33 | 93.97 | 1.99 | 92.37 | 2.09 | 42.67 | 7.22 | 43.83 | 5.95 | 45.17 | 2.89 |
| %Forbs | 10.01 | 4.40 | 18.17 | 1.59 | 5.10 | 0.19 | 7.67 | 0.46 | 20.67 | 1.33 | 19.50 | 3.75 | 15.60 | 3.41 |
| **FAUNA** |  |  |  |  |  |  |  |  |  |  |  |  |  |  |
| Total abundance | 1190.44 | 220.31 | 87.00 | 9.02 | 438.89 | 77.17 | 348.78 | 82.63 | 133.67 | 27.05 | 123.67 | 14.31 | 105.50 | 12.51 |
| Abundance excluding Symphypleona | 348.33 | 32.97 | 86.67 | 9.17 | 291.56 | 21.74 | 257.44 | 61.27 | 133.50 | 27.19 | 120.56 | 16.34 | 105.50 | 12.51 |
| Family richness | 15.67 | 0.33 | 5.67 | 1.20 | 16.67 | 1.76 | 17.00 | 2.31 | 3.67 | 0.67 | 6.00 | 0.58 | 2.33 | 0.33 |
| Shannon index | 0.86 | 0.24 | 1.56 | 0.19 | 1.50 | 0.22 | 1.65 | 0.19 | 1.19 | 0.11 | 1.48 | 0.14 | 0.72 | 0.03 |
| O. Araneae | 9.89 | 3.95 | 0.33 | 0.19 | 6.56 | 2.23 | 6.33 | 0.69 | 0.00 | 0.00 | 0.22 | 0.11 | 0.00 | 0.00 |
| Fam. Linyphiidae | 7.67 | 2.78 | 0.22 | 0.11 | 4.78 | 2.12 | 4.44 | 0.40 | 0.00 | 0.00 | 0.11 | 0.11 | 0.00 | 0.00 |
| O. Coleoptera | 1.33 | 0.38 | 0.00 | 0.00 | 3.56 | 2.06 | 3.00 | 1.15 | 0.00 | 0.00 | 0.33 | 0.00 | 0.00 | 0.00 |
| Fam. Cantharidae | 0.00 | 0.00 | 0.00 | 0.00 | 0.00 | 0.00 | 0.00 | 0.00 | 0.00 | 0.00 | 0.00 | 0.00 | 0.00 | 0.00 |
| O. Diptera | 95.44 | 20.12 | 82.44 | 10.08 | 94.78 | 13.69 | 87.22 | 22.96 | 128.33 | 29.40 | 113.11 | 18.41 | 100.67 | 9.18 |
| Fam. Syrphidae | 0.67 | 0.38 | 0.00 | 0.00 | 0.33 | 0.33 | 0.44 | 0.29 | 0.00 | 0.00 | 0.00 | 0.00 | 0.00 | 0.00 |
| Fam. Tipulidae | 11.44 | 4.78 | 0.44 | 0.22 | 10.78 | 2.41 | 7.67 | 1.39 | 0.50 | 0.29 | 0.22 | 0.22 | 0.00 | 0.00 |
| O. Hemiptera | 79.56 | 12.88 | 2.22 | 0.73 | 86.22 | 15.97 | 62.78 | 21.44 | 2.33 | 1.17 | 3.56 | 1.09 | 3.33 | 2.33 |
| Fam. Cicadellidae | 2.56 | 0.91 | 1.11 | 0.22 | 4.33 | 2.01 | 2.78 | 1.31 | 1.00 | 0.58 | 1.44 | 0.22 | 1.17 | 0.44 |
| Fam. Miridae | 26.22 | 7.23 | 0.11 | 0.11 | 16.56 | 3.78 | 11.00 | 2.52 | 0.00 | 0.00 | 0.00 | 0.00 | 0.00 | 0.00 |
| Fam. Delphacidae | 1.56 | 0.78 | 0.00 | 0.00 | 3.44 | 1.49 | 3.78 | 1.83 | 0.00 | 0.00 | 0.00 | 0.00 | 0.00 | 0.00 |
| Fam. Aphididae | 47.22 | 5.65 | 0.89 | 0.59 | 59.78 | 13.31 | 43.56 | 16.00 | 1.33 | 0.60 | 2.00 | 1.15 | 1.83 | 1.83 |
| O. Hymenoptera | 78.56 | 9.53 | 1.22 | 0.91 | 72.56 | 5.79 | 65.44 | 16.95 | 2.33 | 1.09 | 2.22 | 0.22 | 1.00 | 0.50 |
| O. Symphypleona | 842.11 | 250.07 | 0.33 | 0.33 | 147.33 | 70.17 | 91.33 | 36.17 | 0.17 | 0.17 | 3.11 | 2.19 | 0.00 | 0.00 |

**Table S2** Abbreviations for families/taxa included in multivariate analyses.

| Family/Taxa | Abbreviation |
| --- | --- |
| Acrididae | Ac |
| Anthicidae | An |
| Anthocoridae | At |
| Aphidoidea | Ah |
| Apidae | Ai |
| Araneidae | Ar |
| Cantharidae | Cn |
| Carabidae | Cr |
| Cercopidae | Ce |
| Chrysomelidae | Ch |
| Chrysopidae | Cs |
| Cicadellidae | Ci |
| Cixiidae | Cx |
| Coccinellidae | Co |
| Curculionidae | Cu |
| Delphacidae | De |
| Hydrophiliidae | Hy |
| Kateridae | Ka |
| Lathridiidae | Lat |
| Linyphiidae | Li |
| Lycosidae | Lc |
|  |  |
| Family/Taxa | Abbreviation |
| Lygaeidae | Lg |
| Miridae | Mi |
| Nabidae | Na |
| Nitidulidae | Ni |
| Phalangiidae | Ph |
| Psyllidae | Ps |
| Ptiliidae | Pti |
| Rhagionidae | Rh |
| Scatophagidae | Sa |
| Scolytidae | So |
| Silphidae | Si |
| Sminthuridae | Sm |
| Staphylinidae | St |
| Stratiomyidae | Sr |
| Syrphidae | Sy |
| Tabanidae | Ta |
| Tetragnathidae | Tet |
| Theridiidae | Th |
| Thomisidae | To |
| Thysanoptera | Ty |
| Tingidae | Ti |
| Tipulidae | Tp |
